# Estimation type results related to Fejér inequality with applications

**DOI:** 10.1186/s13660-018-1677-z

**Published:** 2018-04-13

**Authors:** M. Rostamian Delavar, S. S. Dragomir, M. De La Sen

**Affiliations:** 1 Department of Mathematics, Faculty of Basic Sciences, University of Bojnord, Bojnord, Iran; 2 Mathematics, College of Engineering & Science, Victoria University, Melbourne City, Australia; 3 Institute of Research and Development of Processes, University of Basque Country, Bilbao, Spain

**Keywords:** 26A51, 26D15, 52A01, Convex function, Fejér inequality, Trapezoidal formula, Random variable

## Abstract

This paper deals with some new theorems and inequalities about a Fejér type integral inequality which estimate the difference between the right and middle part in Fejér inequality with new bounds. Also some applications to higher moments of random variables, an error estimate for trapezoidal formula, and some inequalities in connection with special means are given.

## Introduction and preliminaries

Throughout the paper, we use an interval $I\subseteq\mathbb {R}$ with the nonempty interior $I^{\circ}$.

The Fejér integral inequality for convex functions has been proved in [1]:

### Theorem 1.1

*Let* $f:[a, b]\to\mathbb{R}$ *be a convex function*. *Then*

1.1 $$\begin{aligned} f\biggl(\frac{a + b}{2}\biggr) \int_{a}^{b} g(x)\,dx&\leq \int _{a}^{b}f(x)g(x)\,dx \\ &\leq\frac{f(a) + f(b)}{2} \int_{a}^{b}g(x)\,dx, \end{aligned}$$

*where* $g: [a, b]\to\mathbb{R}^{+}=[0,+\infty)$ *is integrable and symmetric to* $x=\frac{a+b}{2}\ (g(x)=g(a+b-x), \forall x\in [a,b])$.

To see more results and generalizations about Fejér inequality, we refer the readers to [2–9] and the references therein.

An interesting problem in (1.1) is the estimation of difference for the right-middle part of this inequality which is named in this work as Fejér trapezoidal inequality. In [10], the Fejér trapezoidal inequality related to convex functions has been obtained as follows.

### Theorem 1.2

*Let* $f: I^{\circ}\subseteq\mathbb{R}\to\mathbb{R}$ *be a differentiable mapping*, *where* $a, b\in I$ *with* $a < b$, *and let* $g: [a, b]\to[0,\infty)$ *be a continuous positive mapping symmetric to* $\frac{a+b}{2}$. *If the mapping* $\vert f ' \vert $ *is convex on* $[a, b]$, *then the following inequality holds*:

1.2 $$\begin{aligned} & \biggl\vert \frac{f(a)+f(b)}{2} \int_{a}^{b}g(x)\,dx- \int_{a}^{b} f(x)g(x)\,dx \biggr\vert \\ &\quad \leq\frac{(b-a)}{4}\bigl[ \bigl\vert f'(a) \bigr\vert + \bigl\vert f'(b) \bigr\vert \bigr] \int _{0}^{1} \int_{\frac{1+t}{2}a+\frac{1-t}{2}b}^{\frac{1-t}{2}a+\frac {1+t}{2}b}g(x)\,dx\,dt. \end{aligned}$$

Also, the following theorem was proved in [11]. It estimates the difference between the right and middle part of (1.1) using Hölder’s inequality.

### Theorem 1.3

*Let* $f: I^{\circ}\subseteq\mathbb{R} \to\mathbb{R}$ *be a differentiable mapping*, $a, b \in I^{\circ}$ *with* $a < b$, *and* $w: [a, b] \to\mathbb{R}^{+}$ *be a differentiable mapping symmetric to* $\frac{a+b}{2}$. *If* $\vert f' \vert ^{q}$ *is convex on* $[a,b]$, $q > 1$, *then the following inequality holds*:

1.3 $$\begin{aligned} &\biggl\vert \frac{1}{b-a}\frac{f (a)+f(b)}{2} \int_{a}^{b} w(x)\,dx-\frac {1}{b-a} \int_{a}^{b} f(x)w(x)\,dx \biggr\vert \\ &\quad \leq \frac{1}{2}\biggl[ \int _{0}^{1}\bigl(g(x)\bigr)^{p}\,dt \biggr]^{1\over p}\biggl(\frac{ \vert f'(a) \vert ^{q} + \vert f'(b) \vert ^{q}}{2}\biggr)^{1\over q}, \end{aligned}$$

*where* $\frac{1}{p}+\frac{1}{q}=1$ *and*

$$\begin{aligned} &g(x) = \biggl\vert \int^{b-(b-a)t}_{a+(b-a)t}w(x)\,dx \biggr\vert \end{aligned}$$

*for* $t \in[0, 1]$.

Motivated by the above-mentioned results, in this work we obtain a new trapezoidal form of Fejér inequality which is different from (1.2) and (1.3). To obtain the main result, we assume that the absolute value of the derivative of the considered function is convex. In what follows, we replace this assumption with the boundedness of the derivative and with a Lipschitzian condition for the derivative of the considered function to obtain new estimation type results. Furthermore, some applications in connection with random variable, trapezoidal formula, and special means are given.

The following lemma holds for symmetric functions as well and will be used to obtain various inequalities in the next sections.

### Lemma 1.4

*Suppose that* $w:[a,b]\to\mathbb{R}$ *is an integrable function on* $[a,b]$ *symmetric to* $\frac{a+b}{2}$. *Then*

(i) *for any* $0\leq t\leq\frac{1}{2}$,
  1.4 $$\begin{aligned} \int_{t}^{1} w\bigl(sa+(1-s)b\bigr)\,ds- \int_{0}^{t} w\bigl(sa+(1-s)b \bigr)\,ds=2 \int_{t}^{1\over 2} w\bigl(sa+(1-s)b\bigr)\,ds; \end{aligned}$$
(ii) *for any* $\frac{1}{2}\leq t\leq1$,
  1.5 $$\begin{aligned} \int_{0}^{t} w\bigl(sa+(1-s)b\bigr)\,ds- \int_{t}^{1} w\bigl(sa+(1-s)b \bigr)\,ds=2 \int_{1\over 2}^{t} w\bigl(sa+(1-s)b\bigr)\,ds. \end{aligned}$$

### Proof

(i) Using the change of variable $x=sa+(1-s)b$ for $0\leq t\leq{1\over 2}$, we get

$$\begin{aligned} \int_{t}^{1} w\bigl(sa+(1-s)b\bigr)\,ds- \int_{0}^{t} w\bigl(sa+(1-s)b \bigr)\,ds= \frac{1}{b-a}\biggl[ \int_{a}^{u} w(x)\,dx- \int_{u}^{b} w(x)\,dx\biggr], \end{aligned}$$

where $\frac{a+b}{2}\leq u\leq b$.

Since *w* is symmetric to $\frac{a+b}{2}$, we have

$$\begin{aligned} \int_{{a+b}\over 2}^{b} w(x)\,dx= \int_{a}^{{a+b}\over 2} w(x)\,dx. \end{aligned}$$

Then

$$\begin{aligned} \int_{a}^{u} w(x)\,dx= \int_{a}^{{a+b}\over 2} w(x)\,dx+ \int_{{a+b}\over 2}^{u} w(x)\,dx= \int_{{a+b}\over 2}^{b} w(x)\,dx+ \int_{{a+b}\over 2}^{u} w(x)\,dx. \end{aligned}$$

Also

$$\begin{aligned} \int_{{a+b}\over 2}^{b} w(x)\,dx= \int_{{a+b}\over 2}^{u} w(x)\,dx+ \int_{u}^{b} w(x)\,dx. \end{aligned}$$

So

$$\begin{aligned} \frac{1}{b-a}\biggl[ \int_{a}^{u} w(x)\,dx- \int_{u}^{b} w(x)\,dx\biggr]=\frac {2}{b-a} \int_{{a+b}\over 2}^{u} w(x)\,dx=2 \int_{t}^{{1}\over 2} w \bigl(sa+(1-s)b\bigr)\,ds, \end{aligned}$$

which implies (1.4).

(ii) With the same argument as that used in (i), we can derive (1.5). □

### Remark 1.5

With the assumptions of Lemma 1.4, if *w* is a nonnegative function, then we have the following inequalities:

$$\begin{aligned} \int_{t}^{1} w\bigl(sa+(1-s)b\bigr)\,ds- \int_{0}^{t} w\bigl(sa+(1-s)b \bigr)\,ds\geq0,\quad 0\leq t \leq\frac{1}{2}, \end{aligned}$$

and

$$\begin{aligned} \int_{0}^{t} w\bigl(sa+(1-s)b\bigr)\,ds- \int_{t}^{1} w\bigl(sa+(1-s)b \bigr)\,ds\geq0,\quad \frac{1}{2}\leq t\leq1. \end{aligned}$$

The following identity was obtained in [11] and will be used to obtain the main result.

### Lemma 1.6

*Let* $f: I^{\circ}\subseteq\mathbb{R}\to \mathbb{R}$ *be a differentiable mapping*, $a, b\in I^{\circ}$ *with* $a < b$, *and* $w:[a,b]\to\mathbb{R}^{+}$ *be a differentiable mapping*. *If* $f'\in L[a, b]$, *then the following equality holds*:

1.6 $$\begin{aligned} &\frac{1}{b-a}\biggl(\frac{f(a)+f(b)}{2} \int_{a}^{b}w(x)\,dx- \int_{a}^{b} f(x)w(x)\,dx\biggr) \\ &\quad =\frac{b-a}{2} \int_{0}^{1}p(t)f'\bigl(ta+(1-t)b \bigr)\,dt, \end{aligned}$$

*where*

$$p(t)= \int_{t}^{1} w\bigl(sa+(1-s)b\bigr)\,ds- \int_{0}^{t} w\bigl(sa+(1-s)b\bigr)\,ds. $$

## Main results

For the main result, by using Lemma 1.4, Remark 1.5, and Lemma 1.6, we estimate the difference between the right and middle part of (1.1) with a simple and new face without need of using Hölder’s inequality in the proof.

### Theorem 2.1

*Let* $f:I\to\mathbb{R}$ *be a mapping that is differentiable on* $I^{\circ}$, *let* $a,b\in I^{\circ}$ *be points with* $a < b$, *and let* $w:[a,b]\to\mathbb{R}$ *be a nonnegative integrable mapping that is differentiable on* $(a, b)$. *If w is symmetric to* $\frac{a+b}{2}$, *and if* $\vert f' \vert $ *is convex on* $[a,b]$, *then*

2.1 $$\begin{aligned} &\biggl\vert \frac{f(a)+f(b)}{2} \int_{a}^{b}w(x)\,dx- \int_{a}^{b} f(x)w(x)\,dx \biggr\vert \\ &\quad \leq\bigl[ \bigl\vert f'(a) \bigr\vert + \bigl\vert f'(b) \bigr\vert \bigr] \int_{\frac{a+b}{2}}^{b}w(x) (b-x)\,dx. \end{aligned}$$

### Proof

From Lemma 1.6, Corollary 1.5, and the convexity of $\vert f' \vert $, we have

$$\begin{aligned} & \biggl\vert \frac{f(a)+f(b)}{2} \int_{a}^{b}w(x)\,dx- \int_{a}^{b} f(x)w(x)\,dx \biggr\vert \\ &\quad=\frac{(b-a)^{2}}{2} \biggl\vert \int_{0}^{1}\biggl[ \int_{t}^{1}w \bigl(sa+(1-s)b\bigr)\,ds- \int_{0}^{t}w\bigl(sa+(1-s)b\bigr)\,ds \biggr]f'\bigl(ta+(1-t)b\bigr)\,dt \biggr\vert \\ &\quad\leq\frac{(b-a)^{2}}{2}\biggl\{ \int_{0}^{1\over 2} \biggl\vert \int_{t}^{1} w\bigl(sa+(1-s)b\bigr)\,ds- \int_{0}^{t}w\bigl(sa+(1-s)b\bigr)\,ds \biggr\vert \\ &\qquad{}\times \bigl\vert f' \bigr\vert \bigl(ta+(1-t)b\bigr)\,dt \\ &\qquad{}+ \int_{1\over 2}^{1} \biggl\vert \int_{t}^{1} w\bigl(sa+(1-s)b\bigr)\,ds- \int _{0}^{t}w\bigl(sa+(1-s)b\bigr)\,ds \biggr\vert \bigl\vert f' \bigr\vert \bigl(ta+(1-t)b\bigr)\,dt\biggr\} \\ &\quad\leq\frac{(b-a)^{2}}{2}\biggl\{ \int_{0}^{1\over 2}\biggl( \int_{t}^{1} w\bigl(sa+(1-s)b\bigr)\,ds- \int_{0}^{t}w\bigl(sa+(1-s)b\bigr)\,ds\biggr)\\ &\qquad{}\times \bigl(t \bigl\vert f'(a) \bigr\vert +(1-t) \bigl\vert f'(b) \bigr\vert \bigr)\,dt \\ &\qquad{}+ \int_{1\over 2}^{1}\biggl( \int_{0}^{t} w\bigl(sa+(1-s)b\bigr)\,ds- \int_{t}^{1} w\bigl(sa+(1-s)b\bigr)\,ds\biggr) \bigl(t \bigl\vert f'(a) \bigr\vert +(1-t) \bigl\vert f'(b) \bigr\vert \bigr)\biggr\} \\ &\quad=J. \end{aligned}$$

If we change the order of integration in *J*, then

$$\begin{aligned} J={}&\frac{(b-a)^{2}}{2}\biggl\{ \int_{0}^{1\over 2} \int_{0}^{s} w \bigl(sa+(1-s)b\bigr) \bigl(t \bigl\vert f'(a) \bigr\vert +(1-t) \bigl\vert f'(b) \bigr\vert \bigr)\,dt\,ds \\ &{}+ \int_{1\over 2}^{1} \int_{0}^{1\over 2} w\bigl(sa+(1-s)b\bigr) \bigl(t \bigl\vert f'(a) \bigr\vert +(1-t) \bigl\vert f'(b) \bigr\vert \bigr)\,dt\,ds \\ &{}- \int_{0}^{1\over 2} \int_{s}^{1\over 2} w\bigl(sa+(1-s)b\bigr) \bigl(t \bigl\vert f'(a) \bigr\vert +(1-t) \bigl\vert f'(b) \bigr\vert \bigr)\,dt\,ds \\ &{}+ \int_{1\over 2}^{1} \int_{s}^{1} w\bigl(sa+(1-s)b\bigr) \bigl(t \bigl\vert f'(a) \bigr\vert +(1-t) \bigl\vert f'(b) \bigr\vert \bigr)\,dt\,ds \\ &{}+ \int_{0}^{1\over 2} \int_{1\over 2}^{1} w\bigl(sa+(1-s)b\bigr) \bigl(t \bigl\vert f'(a) \bigr\vert +(1-t) \bigl\vert f'(b) \bigr\vert \bigr)\,dt\,ds \\ &{}- \int_{1\over 2}^{1} \int_{1\over 2}^{s} w\bigl(sa+(1-s)b\bigr) \bigl(t \bigl\vert f'(a) \bigr\vert +(1-t) \bigl\vert f'(b) \bigr\vert \bigr)\,dt\,ds\biggr\} . \end{aligned}$$

Calculating all inner integrals in *J*, we get

$$\begin{aligned} J={}&\frac{(b-a)^{2}}{2}\biggl\{ \int_{0}^{1\over 2} w\bigl(sa+(1-s)b \bigr) \biggl( {1\over 2}s^{2} \bigl\vert f'(a) \bigr\vert +\biggl(s-{1\over 2}s^{2}\biggr) \bigl\vert f'(b) \bigr\vert \biggr)\,ds \\ &{}+ \int_{1\over 2}^{1} w\bigl(sa+(1-s)b\bigr) \biggl( {1\over 8} \bigl\vert f'(a) \bigr\vert + {3\over 8} \bigl\vert f'(b) \bigr\vert \biggr)\,ds \\ &{}- \int_{0}^{1\over 2} w\bigl(sa+(1-s)b\bigr) \biggl(\biggl( {1\over 8}-{1\over 2}s^{2}\biggr) \bigl\vert f'(a) \bigr\vert +\biggl({3\over 8}-s+ {1\over 2}s^{2}\biggr) \bigl\vert f'(b) \bigr\vert \biggr)\,ds \\ &{}+ \int_{1\over 2}^{1} w\bigl(sa+(1-s)b\bigr) \biggl(\biggl( {1\over 2}-{1\over 2}s^{2}\biggr) \bigl\vert f'(a) \bigr\vert +\biggl({1\over 2}-s+ {1\over 2}s^{2}\biggr) \bigl\vert f'(b) \bigr\vert \biggr)\,ds \\ &{}+ \int_{0}^{1\over 2} w\bigl(sa+(1-s)b\bigr) \biggl( {3\over 8} \bigl\vert f'(a) \bigr\vert + {1\over 8} \bigl\vert f'(b) \bigr\vert \biggr)\,ds \\ &{}- \int_{1\over 2}^{1} w\bigl(sa+(1-s)b\bigr) \biggl(\biggl( {1\over 2}s^{2}-{1\over 8}\biggr) \bigl\vert f'(a) \bigr\vert +\biggl(s-{1\over 2}s^{2}- {3\over 8}\biggr) \bigl\vert f'(b) \bigr\vert \biggr)\,ds\biggr\} . \end{aligned}$$

A simple form of *J* can be obtained as follows:

$$\begin{aligned} J={}&\frac{(b-a)^{2}}{2}\biggl\{ \int_{0}^{1\over 2} w\bigl(sa+(1-s)b \bigr) \biggl( \biggl(s^{2}+{1\over 4}\biggr) \bigl\vert f'(a) \bigr\vert +\biggl(-s^{2}+2s- {1\over 4}\biggr) \bigl\vert f'(b) \bigr\vert \biggr)\,ds \\ &{}+ \int_{1\over 2}^{1} w\bigl(sa+(1-s)b\bigr) \biggl( \biggl(-s^{2}+{3\over 4}\biggr) \bigl\vert f'(a) \bigr\vert +\biggl(s^{2}-2s+{5\over 4} \biggr) \bigl\vert f'(b) \bigr\vert \biggr)\,ds\biggr\} . \end{aligned}$$

If we use the change of variable $x=sa+(1-s)b$ in *J*, then

$$\begin{aligned} J={}&\frac{(b-a)}{2}\biggl\{ \int_{\frac{a+b}{2}}^{b} w(x) \biggl( \biggl[\biggl( \frac{x-b}{a-b}\biggr)^{2}+{1\over 4}\biggr] \bigl\vert f'(a) \bigr\vert \\ &{} +\biggl[2\biggl(\frac {x-b}{a-b} \biggr)-\biggl(\frac{x-b}{a-b}\biggr)^{2}-{1\over 4} \biggr] \bigl\vert f'(b) \bigr\vert \biggr)\,dx \\ &{}+ \int_{a}^{\frac{a+b}{2}} w(x) \biggl(\biggl[ {3\over 4}-\biggl(\frac {x-b}{a-b}\biggr)^{2}\biggr] \bigl\vert f'(a) \bigr\vert \\ &{}+\biggl[\biggl(\frac{x-b}{a-b} \biggr)^{2}-2\biggl(\frac {x-b}{a-b}\biggr)+{5\over 4} \biggr] \bigl\vert f'(b) \bigr\vert \biggr)\,dx\biggr\} . \end{aligned}$$

On the other hand, since *w* is symmetric to $\frac{a+b}{2}$, we have

$$\begin{aligned} & \int_{a}^{\frac{a+b}{2}} w(x) \biggl(\biggl[ {3\over 4}-\biggl(\frac {x-b}{a-b}\biggr)^{2}\biggr] \bigl\vert f'(a) \bigr\vert +\biggl[\biggl(\frac{x-b}{a-b} \biggr)^{2}-2\biggl(\frac {x-b}{a-b}\biggr)+{5\over 4} \biggr] \bigl\vert f'(b) \bigr\vert \biggr)\,dx \\ &\quad = \int_{\frac{a+b}{2}}^{b} w(x) \biggl(\biggl[ {3\over 4}-\biggl(\frac {a-x}{a-b}\biggr)^{2}\biggr] \bigl\vert f'(a) \bigr\vert +\biggl[\biggl(\frac{a-x}{a-b} \biggr)^{2}-2\biggl(\frac {a-x}{a-b}\biggr)+{5\over 4} \biggr] \bigl\vert f'(b) \bigr\vert \biggr)\,dx. \end{aligned}$$

So

$$\begin{aligned} J={}&\frac{(b-a)}{2}\biggl\{ \int_{\frac{a+b}{2}}^{b} w(x) \biggl( \biggl[1+\biggl( \frac{x-b}{a-b}\biggr)^{2}-\biggl(\frac{a-x}{a-b} \biggr)^{2}\biggr] \bigl\vert f'(a) \bigr\vert \\ &{}+\biggl[1-2\biggl(\frac{a-x}{a-b}\biggr)-\biggl(\frac{x-b}{a-b} \biggr)^{2}+\biggl(\frac {a-x}{a-b}\biggr)^{2}+2\biggl( \frac{x-b}{a-b}\biggr)\biggr] \bigl\vert f'(b) \bigr\vert \biggr)\,dx\biggr\} \\ ={}&\frac{(b-a)}{2}\biggl\{ \int_{\frac{a+b}{2}}^{b} w(x) \biggl(2 \biggl[ \frac{x-b}{a-b}\biggr] \bigl\vert f'(a) \bigr\vert +2\biggl[ \frac{x-b}{a-b} \biggr] \bigl\vert f'(b) \bigr\vert \biggr)\,dx\biggr\} \\ ={}&\bigl( \bigl\vert f'(a) \bigr\vert + \bigl\vert f'(b) \bigr\vert \bigr) \int_{\frac{a+b}{2}}^{b} w(x) (b-x)\,dx. \end{aligned}$$

 □

### Remark 2.2

We can obtain another form of (1.6) in Lemma 1.6. In fact we get

2.2 $$\begin{aligned} &\frac{1}{b-a}\biggl(\frac{f(a)+f(b)}{2} \int_{a}^{b}g(x)\,dx- \int_{a}^{b} f(x)g(x)\,dx\biggr) \\ &\quad =\frac{b-a}{2} \int_{0}^{1}q(t)f'\bigl(tb+(1-t)a \bigr)\,dt, \end{aligned}$$

where $q(t)=p(1-t)=-p(t)\leq0$.

Now using (2.2) in the proof of Theorem 2.1 implies another form of (2.1).

2.3 $$\begin{aligned} &\biggl\vert \frac{f(a)+f(b)}{2} \int_{a}^{b}w(x)\,dx- \int_{a}^{b} f(x)w(x)\,dx \biggr\vert \\ &\quad \leq\bigl[ \bigl\vert f'(a) \bigr\vert + \bigl\vert f'(b) \bigr\vert \bigr] \int_{a}^{\frac{a+b}{2}}w(x) (x-a)\,dx. \end{aligned}$$

### Corollary 2.3

(Theorem 2.2 in [12])

*If in* (2.1) *and* (2.3) *we consider* $w\equiv1$, *then*

$$\begin{aligned} \biggl\vert \frac{f(a)+f(b)}{2}(b-a)- \int_{a}^{b} f(x)\,dx \biggr\vert \leq \frac {(b-a)^{2}}{8}\bigl( \bigl\vert f'(a) \bigr\vert + \bigl\vert f'(b) \bigr\vert \bigr). \end{aligned}$$

## Further estimation results

It is known that any convex function defined on the interval $[a,b]$ is bounded and satisfies a Lipschitz condition [13]. So in this section instead of the convexity of derivative we consider the boundedness of the derivative and a Lipschitzian condition for the derivative of the considered function respectively to obtain new estimation type results.

Now suppose that the derivative of the considered function is bounded from below and above. Then we can derive an estimation type result related to Fejér inequality.

### Theorem 3.1

*Let* $f:I\to\mathbb{R}$ *be a mapping that is differentiable on* $I^{\circ}$, *let* $a,b\in I^{\circ}$ *be points with* $a < b$, *and let* $w:[a,b]\to\mathbb{R}$ *be a nonnegative integrable mapping that is differentiable on* $(a, b)$. *Assume that* $f'$ *is integrable on* $[a,b]$ *and there exist constants* $m< M$ *such that*

$$-\infty< m\leq f'(x)\leq M< \infty\quad \textit{for all } x\in[a,b]. $$

*Then*

3.1 $$\begin{aligned} & \biggl\vert \frac{f(a)+f(b)}{2(b-a)} \int_{a}^{b}w(x)\,dx-\frac{1}{b-a} \int _{a}^{b} f(x)w(x)\,dx-\frac{m+M}{4} \int_{0}^{1}p(t)\,dt \biggr\vert \\ &\quad\leq\frac{(M-m)(b-a)}{4} \int_{0}^{1} \bigl\vert p(t) \bigr\vert \,dt, \end{aligned}$$

*where* $p(t)$ *is defined in Lemma *1.6.

### Proof

From Lemma 1.6 we have

$$\begin{aligned} &\frac{1}{b-a}\biggl(\frac{f(a)+f(b)}{2} \int_{a}^{b}w(x)\,dx- \int_{a}^{b} f(x)w(x)\,dx\biggr) \\ &\quad=\frac{b-a}{2} \int_{0}^{1}p(t)\biggl[f' \bigl(ta+(1-t)b\bigr)-\frac {m+M}{2}+\frac{m+M}{2}\biggr]\,dt \\ &\quad=\frac{(m+M)(b-a)}{4} \int_{0}^{1}p(t)\,dt+\frac{b-a}{2} \int _{0}^{1}p(t)\biggl[f' \bigl(ta+(1-t)b\bigr)-\frac{m+M}{2}\biggr]\,dt. \end{aligned}$$

So

$$\begin{aligned} J&=\frac{1}{b-a}\biggl(\frac{f(a)+f(b)}{2} \int_{a}^{b}w(x)\,dx- \int_{a}^{b} f(x)w(x)\,dx\biggr)-\frac{(m+M)(b-a)}{4} \int_{0}^{1}p(t)\,dt \\ &=\frac{b-a}{2} \int_{0}^{1}p(t)\biggl[f' \bigl(ta+(1-t)b\bigr)-\frac {m+M}{2}\biggr]\,dt. \end{aligned}$$

Therefore

$$\begin{aligned} \vert J \vert &\leq\frac{b-a}{2} \int_{0}^{1} \bigl\vert p(t) \bigr\vert \biggl\vert f'\bigl(ta+(1-t)b \bigr)-\frac{m+M}{2} \biggr\vert \,dt \\ &\leq\frac{(M-m)(b-a)}{4} \int_{0}^{1} \bigl\vert p(t) \bigr\vert \,dt, \end{aligned}$$

since from the inequality $m\leq f'(ta+(1-t)b)\leq M$, we have

$$m-\frac{m+M}{2}\leq f'\bigl(ta+(1-t)b\bigr)- \frac{m+M}{2}\leq M-\frac {m+M}{2}, $$

which implies that

$$\biggl\vert f'\bigl(ta+(1-t)b\bigr)-\frac{m+M}{2} \biggr\vert \leq\frac{M-m}{2}. $$

 □

### Remark 3.2

If in Theorem 3.1 we assume that *w* is symmetric to $\frac{a+b}{2}$, then from Lemma 1.4 we have

$$\begin{aligned} \int_{0}^{1} \bigl\vert p(t) \bigr\vert \,dt&=2 \int_{0}^{1} \biggl\vert \int_{t}^{1\over 2}w \bigl(sa+(1-s)b\bigr)\,ds \biggr\vert \,dt\\ &\leq2 \int_{0}^{1} \biggl\vert t-{1 \over 2} \biggr\vert \sup_{s\in[t,\frac{1}{2}]} \bigl\vert w\bigl(sa+(1-s)b\bigr) \bigr\vert \,dt\leq\frac {1}{2} \Vert w \Vert _{\infty}. \end{aligned}$$

Then

$$\begin{aligned} & \biggl\vert \frac{f(a)+f(b)}{2(b-a)} \int_{a}^{b}w(x)\,dx-\frac{1}{b-a} \int _{a}^{b} f(x)w(x)\,dx-\frac{m+M}{4} \int_{0}^{1}p(t)\,dt \biggr\vert \\ &\quad \leq\frac{(M-m)(b-a)}{8} \Vert w \Vert _{\infty}. \end{aligned}$$

Also, using Hölder’s inequality, we have

$$\begin{aligned} \int_{0}^{1} \bigl\vert p(t) \bigr\vert \,dt&\leq2 \int_{0}^{1} \biggl\vert t-{1 \over 2} \biggr\vert ^{1\over p}\biggl( \int_{t}^{1\over 2} \bigl\vert w\bigl(sa+(1-s)b\bigr) \bigr\vert ^{q} ds\biggr)^{1\over q}\,dt\\ &\leq2 \Vert w \Vert _{q} \int_{0}^{1} \biggl\vert t-{1 \over 2} \biggr\vert ^{1\over p}\,dt, \end{aligned}$$

which implies that

$$\begin{aligned} & \biggl\vert \frac{f(a)+f(b)}{2(b-a)} \int_{a}^{b}w(x)\,dx-\frac{1}{b-a} \int _{a}^{b} f(x)w(x)\,dx-\frac{m+M}{4} \int_{0}^{1}p(t)\,dt \biggr\vert \\ &\quad\leq\frac{(M-m)(b-a)}{2} \Vert w \Vert _{q} \int_{0}^{1} \biggl\vert t-{1 \over 2} \biggr\vert ^{1\over p}\,dt. \end{aligned}$$

### Corollary 3.3

*In Theorem *3.1 *if* $w\equiv1$, *then*

$$\begin{aligned} \biggl\vert \frac{f(a)+f(b)}{2}-\frac{1}{b-a} \int_{a}^{b} f(x)\,dx \biggr\vert \leq \frac{m(1+a-b)+M(1+b-a)}{8}. \end{aligned}$$

### Proof

If we consider $w\equiv1$, then the relations $\Vert w \Vert _{\infty}=1$ and $\int_{0}^{1} \vert p(t) \vert \,dt\leq{1\over 2}$ imply that

$$\begin{aligned} &\frac{1}{b-a} \biggl\vert \frac{f(a)+f(b)}{2}(b-a)- \int_{a}^{b} f(x)\,dx \biggr\vert \\ &\quad\leq \biggl\vert \frac{m+M}{4} \int_{0}^{1}p(t)\,dt \biggr\vert +\frac {(M-m)(b-a)}{8} \\ &\quad\leq\frac{m+M}{8}+\frac{(M-m)(b-a)}{8}=\frac{m(1+a-b)+M(1+b-a)}{8}. \end{aligned}$$

 □

Estimation for difference between the right and middle terms of (1.1) when the derivative of the function satisfies a Lipschitz condition is our next aim.

### Definition 3.4

([13])

A function $f:[a,b]\rightarrow\mathbb{R}$ is said to satisfy Lipschitz condition on $[a,b]$ if there is a constant *K* so that, for any two points $x,y\in{}[ a,b]$,

$$\bigl\vert f(x)-f(y)\bigr\vert \leq K \vert x-y \vert . $$

### Theorem 3.5

*Let* $f:I\to\mathbb{R}$ *be a mapping that is differentiable on* $I^{\circ}$, *let* $a,b\in I^{\circ}$ *be points with* $a < b$, *and let* $w:[a,b]\to\mathbb{R}$ *be a nonnegative integrable mapping that is differentiable on* $(a, b)$. *Assume that* $f'$ *is integrable on* $[a,b]$ *and satisfies a Lipschitz condition for some* $K>0$. *Then*

3.2 $$\begin{aligned} & \biggl\vert \frac{f(a)+f(b)}{2(b-a)} \int_{a}^{b}w(x)\,dx-\frac{1}{b-a} \int _{a}^{b} f(x)w(x)\,dx-\frac{1}{2}f' \biggl(\frac{a+b}{2}\biggr) \int _{0}^{1}p(t)\,dt \biggr\vert \\ &\quad\leq K\frac{(b-a)^{2}}{2} \int_{0}^{1} \biggl\vert t-{1\over 2} \biggr\vert \bigl\vert p(t) \bigr\vert \,dt, \end{aligned}$$

*where* $p(t)$ *is defined in Lemma *1.6.

### Proof

From Lemma 1.6 we get

$$\begin{aligned} &\frac{1}{b-a}\biggl(\frac{f(a)+f(b)}{2} \int_{a}^{b}w(x)\,dx- \int_{a}^{b} f(x)w(x)\,dx\biggr) \\ &\quad=\frac{b-a}{2} \int_{0}^{1}p(t)\biggl[f' \bigl(ta+(1-t)b\bigr)-f' \biggl(\frac{a+b}{2} \biggr)+f'\biggl(\frac{a+b}{2}\biggr)\biggr]\,dt \\ &\quad=\frac{b-a}{2} \int_{0}^{1}p(t)\biggl[f' \bigl(ta+(1-t)b\bigr)-f' \biggl(\frac{a+b}{2}\biggr)\biggr]\,dt+ \frac{b-a}{2}f'\biggl(\frac{a+b}{2} \biggr) \int_{0}^{1}p(t)\,dt. \end{aligned}$$

Then

$$\begin{aligned} &\frac{1}{b-a}\biggl(\frac{f(a)+f(b)}{2} \int_{a}^{b}w(x)\,dx- \int_{a}^{b} f(x)w(x)\,dx\biggr)-\frac{b-a}{2}f' \biggl(\frac{a+b}{2}\biggr) \int _{0}^{1}p(t)\,dt \\ &\quad=\frac{b-a}{2} \int_{0}^{1}p(t)\biggl[f' \bigl(ta+(1-t)b\bigr)-f' \biggl(\frac{a+b}{2}\biggr)\biggr]\,dt. \end{aligned}$$

Since $f'$ satisfies a Lipschitz condition for some $K>0$, then

$$\begin{aligned} \biggl\vert f'\bigl(ta+(1-t)b\bigr)-f'\biggl( \frac{a+b}{2}\biggr) \biggr\vert \leq K \biggl\vert ta+(1-t)b- \frac{a+b}{2} \biggr\vert =K \biggl\vert t-{1\over 2} \biggr\vert (b-a). \end{aligned}$$

Hence

$$\begin{aligned} & \biggl\vert \frac{f(a)+f(b)}{2(b-a)} \int_{a}^{b}w(x)\,dx-\frac{1}{b-a} \int _{a}^{b} f(x)w(x)\,dx-\frac{1}{2}f' \biggl(\frac{a+b}{2}\biggr) \int _{0}^{1}p(t)\,dt \biggr\vert \\ &\quad\leq K\frac{(b-a)^{2}}{2} \int_{0}^{1} \biggl\vert t-{1\over 2} \biggr\vert \bigl\vert p(t) \bigr\vert \,dt. \end{aligned}$$

 □

### Remark 3.6

In Theorem 3.5 assume that *w* is symmetric to $\frac{a+b}{2}$. With the same argument as in Remark 3.2 and using Lemma 1.4, we get

$$\begin{aligned} & \biggl\vert \frac{f(a)+f(b)}{2(b-a)} \int_{a}^{b}w(x)\,dx-\frac{1}{b-a} \int _{a}^{b} f(x)w(x)\,dx-\frac{1}{2}f' \biggl(\frac{a+b}{2}\biggr) \int _{0}^{1}p(t)\,dt \biggr\vert \\ &\quad \leq K(b-a)^{2} \int_{0}^{1} \int_{t}^{1\over 2} \biggl\vert t-{1\over 2} \biggr\vert \bigl\vert w \bigl(sa+(1-s)b\bigr) \bigr\vert \,ds\,dt. \end{aligned}$$

Also we have

$$\begin{aligned} & \biggl\vert \frac{f(a)+f(b)}{2(b-a)} \int_{a}^{b}w(x)\,dx-\frac{1}{b-a} \int _{a}^{b} f(x)w(x)\,dx-\frac{1}{2}f' \biggl(\frac{a+b}{2}\biggr) \int _{0}^{1}p(t)\,dt \biggr\vert \\ &\quad\leq K(b-a)^{2} \Vert w \Vert _{\infty} \int_{0}^{1} \biggl(t-{1\over 2} \biggr)^{2}\,dt=\frac {K(b-a)^{2}}{12} \Vert w \Vert _{\infty}, \end{aligned}$$

which implies that

$$\begin{aligned} &\biggl\vert \frac{f(a)+f(b)}{2(b-a)} \int_{a}^{b}w(x)\,dx-\frac{1}{b-a} \int_{a}^{b} f(x)w(x)\,dx \biggr\vert \\ &\quad\leq\biggl( \frac{K(b-a)^{2}}{12}+{1\over 4} \biggl\vert f'\biggl( \frac {a+b}{2}\biggr) \biggr\vert \biggr) \Vert w \Vert _{\infty}. \end{aligned}$$

### Corollary 3.7

*In Theorem *3.5 *if* $w\equiv1$, *then*

$$\begin{aligned} \biggl\vert \frac{f(a)+f(b)}{2}-\frac{1}{b-a} \int_{a}^{b} f(x)\,dx \biggr\vert \leq\biggl( \frac{K(b-a)^{2}}{12}+{1\over 4} \biggl\vert f'\biggl( \frac {a+b}{2}\biggr) \biggr\vert \biggr). \end{aligned}$$

## Applications

### Random variable

Suppose that for $0< a< b$, $w:[a,b]\to[0,+\infty)$ is a continuous probability density function related to a continuous random variable *X* which is symmetric about $\frac{a+b}{2}$. Also, for $r\in\mathbb {R}$, suppose that the *r*-moment

$$E_{r}(X)= \int_{a}^{b}x^{r}w(x)\,dx $$

is finite.

(1) If we consider $f(x)=x^{r}$ for $r\geq2$ and $x\in[a,b]$, then $\vert f'(x) \vert =rx^{r-1}$ which is a convex function and so from (2.1) in Theorem 2.1 we have

$$\begin{aligned} & \biggl\vert \frac{a^{r}+b^{r}}{2}-E_{r}(X) \biggr\vert \leq \frac{r(b-a)}{4} \bigl(a^{r-1}+b^{r-1}\bigr), \end{aligned}$$

since

$$\begin{aligned} \biggl\vert \frac{a^{r}+b^{r}}{2}-E_{r}(X) \biggr\vert &\leq r \bigl(a^{r-1}+b^{r-1} \bigr) \int_{\frac{a+b}{2}}^{b}w(x) (b-x)\,dx \\ &\leq r\bigl(a^{r-1}+b^{r-1}\bigr)\frac{b-a}{2} \int_{\frac {a+b}{2}}^{b} w(x)\,dx=r\bigl(a^{r-1}+b^{r-1} \bigr)\frac{b-a}{4}, \end{aligned}$$

where from the fact that *w* is symmetric and $\int_{a}^{b} w(x)\,dx=1$, we have $\int_{\frac{a+b}{2}}^{b} w(x)\,dx={1\over 2}$.

If $r = 1$, $E(X)$ is the expectation of the random variable *X* and from the above inequality, we obtain the following known bound:

4.1 $$\begin{aligned} & \biggl\vert \frac{a+b}{2}-E(X) \biggr\vert \leq \frac{b-a}{2}. \end{aligned}$$

(2) Notice that if *w* is nonnegative, then

$$\begin{aligned} \bigl\vert p(t) \bigr\vert & = \biggl\vert \int_{t}^{1} w\bigl(sa+(1-s)b\bigr)\,ds- \int_{0}^{t} w \bigl(sa+(1-s)b\bigr)\,ds \biggr\vert \\ &\leq \int_{t}^{1} w\bigl(sa+(1-s)b\bigr)\,ds+ \int_{0}^{t} w\bigl(sa+(1-s)b \bigr)\,ds= \int_{0}^{1} w\bigl(sa+(1-s)b\bigr)\,ds=1. \end{aligned}$$

Now if we consider $f(x)=x^{r}$ for $r\in\mathbb{R}$ and $x\in[a,b]$, then $m=ra^{r-1}\leq f'(x)=rx^{r-1}\leq rb^{r-1}=M$, and so from (3.1) in Theorem 3.1 we have

$$\begin{aligned} &\frac{1}{b-a} \biggl\vert \frac{a^{r}+b^{r}}{2}-E_{r}(X)- \frac {r(a^{r-1}+b^{r-1})(b-a)}{4} \int_{0}^{1} p(t) \,dt \biggr\vert \\ &\quad \leq\frac{r(b^{r-1}-a^{r-1})(b-a)}{4} \int_{0}^{1} \bigl\vert p(t) \bigr\vert \,dt\leq \frac {r(b^{r-1}-a^{r-1})(b-a)}{4}. \end{aligned}$$

It follows that

$$\begin{aligned} &\frac{1}{b-a} \biggl\vert \frac{a^{r}+b^{r}}{2}-E_{r}(X) \biggr\vert \\ &\quad \leq\frac {r(b^{r-1}-a^{r-1})(b-a)}{4} +\frac{r(a^{r-1}+b^{r-1})}{4} \int_{0}^{1} \bigl\vert p(t) \bigr\vert \,dt \\ &\quad\leq\frac{r(b^{r-1}-a^{r-1})(b-a)}{4}+\frac{r(a^{r-1}+b^{r-1})}{4}. \end{aligned}$$

Therefore

$$\begin{aligned} & \biggl\vert \frac{a^{r}+b^{r}}{2}-E_{r}(X) \biggr\vert \leq \frac {r(b^{r-1}-a^{r-1})(b-a)^{2}}{4} +\frac{r(a^{r-1}+b^{r-1})(b-a)}{4}. \end{aligned}$$

If we consider $r=1$ in the above inequality, then we recapture (4.1).

(3) If we consider $f(x)=x^{r}$ for $r\in\mathbb{R}$ and $x\in [a,b]$, then the Lipschitz constant $K=\sup_{x\in[a,b]} \vert f'(x) \vert =\sup_{x\in[a,b]}rx^{r-1}$ is equivalent to

$$ K= \textstyle\begin{cases} rb^{r-1}, & \text{$r\geq1$;} \\ ra^{r-1}, & \text{$r< 1$.}\end{cases} $$

So from (3.2) in Theorem 3.5 we have

$$\begin{aligned} & \biggl\vert \frac{a^{r}+b^{r}}{2}-E_{r}(X) \biggr\vert \\ &\quad\leq K \frac{(b-a)^{2}}{2} \int _{0}^{1} \biggl\vert t-{1\over 2} \biggr\vert \bigl\vert p(t) \bigr\vert \,dt+f'\biggl( \frac{a+b}{2}\biggr)\frac {b-a}{2} \int_{0}^{1} \bigl\vert p(t) \bigr\vert \,dt \\ &\quad\leq K\frac{(b-a)^{2}}{2} \int_{0}^{1} \biggl\vert t-{1\over 2} \biggr\vert \,dt+f'\biggl(\frac {a+b}{2}\biggr) \frac{b-a}{2}= K\frac{(b-a)^{2}}{8}+f'\biggl(\frac {a+b}{2} \biggr)\frac{b-a}{2}, \end{aligned}$$

which implies that

$$\begin{aligned} & \biggl\vert \frac{a^{r}+b^{r}}{2}-E_{r}(X) \biggr\vert \leq \textstyle\begin{cases} \frac{r(b-a)}{2}[\frac{b^{r-1}(b-a)^{2}}{4}+(\frac {a+b}{2})^{r-1}], & \text{$r\geq1$;} \\ \frac{r(b-a)}{2}[\frac{a^{r-1}(b-a)^{2}}{4}+(\frac {a+b}{2})^{r-1}], & \text{$r< 1$.}\end{cases}\displaystyle \end{aligned}$$

### Trapezoidal formula

Consider the partition (P) of the interval $[a,b]$ as $a=x_{0}< x_{1}< x_{2}<\cdots<x_{n}=b$. The quadrature formula is

$$\int_{a}^{b}f(x)w(x)\,dx=T(f,w,P)+E(f,w,P), $$

where

$$T(f,w,P)=\sum_{i=0}^{n-1}\frac{f(x_{i})+f(x_{i+1})}{2} \int _{x_{i}}^{x_{i+1}}w(x)\,dx, $$

is the trapezoidal form and $E(f,w,P)$ is the associated approximation error.

For each $i\in\{0,1,\ldots,n-1\}$, consider the interval $[x_{i},x_{i+1}]$ of the partition (P) of the interval $[a,b]$. Suppose that all the conditions of Theorem 2.1 are satisfied on $[x_{i},x_{i+1}]$. Then

4.2 $$\begin{aligned} & \biggl\vert \frac{f(x_{i})+f(x_{i+1})}{2} \int_{x_{i}}^{x_{i+1}}w(x)\,dx- \int _{x_{i}}^{x_{i+1}} f(x)w(x)\,dx \biggr\vert \\ &\quad \leq\bigl[ \bigl\vert f'(x_{i}) \bigr\vert + \bigl\vert f'(x_{i+1}) \bigr\vert \bigr] \int_{\frac {x_{i}+x_{i+1}}{2}}^{x_{i+1}}w(x) (x_{i+1}-x)\,dx. \end{aligned}$$

Now, if all the conditions of Theorem 2.1 are satisfied for the partition (P) on the interval $[a,b]$, then using inequality (4.2), summing with respect to *i* from $i=0$ to $i={n-1}$, and using the triangle inequality, we obtain

$$\begin{aligned} & \biggl\vert T(f,w,P)- \int_{a}^{b}f(x)w(x)\,dx \biggr\vert \\ &\quad = \Biggl\vert \sum_{i=0}^{n-1}\biggl[\frac{f(x_{i})+f(x_{i+1})}{2} \int _{x_{i}}^{x_{i+1}}w(x)\,dx- \int_{x_{i}}^{x_{i+1}} f(x)w(x)\,dx\biggr] \Biggr\vert \\ &\quad\leq\sum_{i=0}^{n-1} \biggl\vert \frac{f(x_{i})+f(x_{i+1})}{2} \int _{x_{i}}^{x_{i+1}}w(x)\,dx- \int_{x_{i}}^{x_{i+1}} f(x)w(x)\,dx \biggr\vert \\ &\quad\leq\sum_{i=0}^{n-1}\bigl[ \bigl\vert f'(x_{i}) \bigr\vert + \bigl\vert f'(x_{i+1}) \bigr\vert \bigr] \int _{\frac{x_{i}+x_{i+1}}{2}}^{x_{i+1}}w(x) (x_{i+1}-x)\,dx. \end{aligned}$$

So we get the error bound:

4.3 $$\begin{aligned} \bigl\vert E(f,w,P) \bigr\vert \leq\sum _{i=0}^{n-1}\bigl[ \bigl\vert f'(x_{i}) \bigr\vert + \bigl\vert f'(x_{i+1}) \bigr\vert \bigr] \int _{\frac{x_{i}+x_{i+1}}{2}}^{x_{i+1}}w(x) (x_{i+1}-x)\,dx. \end{aligned}$$

#### Corollary 4.1

*If* $w\equiv1$ *in* (4.3), *then we recapture the inequality obtained in Proposition *4.1 *in* [12]:

$$\begin{aligned} \bigl\vert E(f,P) \bigr\vert \leq\frac{1}{8}\sum _{i=0}^{n-1} \bigl[ \bigl\vert f'(x_{i}) \bigr\vert + \bigl\vert f'(x_{i+1}) \bigr\vert \bigr](x_{i+1}-x_{i})^{2}. \end{aligned}$$

### Special means

In the literature, the following means for real numbers $a, b\in \mathbb{R}$ are well known:

$$\begin{aligned} &A(a,b)=\frac{a+b}{2} \quad \text{arithmetic mean}, \\ &L_{n}(a,b)=\biggl[\frac{b^{n+1}-a^{n+1}}{(n+1)(b-a)}\biggr]^{\frac {1}{n}}\quad \text{generalized log-mean, $n\in\mathbb{N}, a< b$}. \end{aligned}$$

Consider $f(x)=x^{n}$ for $x>0, n\in\mathbb{N}$ and a differentiable symmetric (to $\frac{a+b}{2}$) mapping $w:[a,b]\to\mathbb{R}^{+}$. Theorem 2.1 implies the following inequality:

$$\begin{aligned} \biggl\vert \frac{a^{n}+b^{n}}{2} \int_{a}^{b}w(x)\,dx- \int_{a}^{b}x^{n}w(x)\,dx \biggr\vert \leq n\bigl( \vert a \vert ^{n-1}+ \vert b \vert ^{n-1} \bigr) \int_{\frac{a+b}{2}}^{b}w(x) (b-x)\,dx. \end{aligned}$$

So

4.4 $$\begin{aligned} & \biggl\vert A\bigl(a^{n},b^{n}\bigr) \int_{a}^{b}w(x)\,dx- \int_{a}^{b}x^{n}w(x)\,dx \biggr\vert \leq 2nA\bigl( \vert a \vert ^{n-1}, \vert b \vert ^{n-1}\bigr) \int_{\frac{a+b}{2}}^{b}w(x) (b-x)\,dx. \end{aligned}$$

If we consider $w\equiv1$ in (4.4), then we recapture the following result.

#### Corollary 4.2

(Proposition 3.1 in [12])

*Let* $a, b \in\mathbb{R}$, $a < b$, *and* $n\in\mathbb{ N}$, $n \geq 2$. *Then the following inequality holds*:

$$\begin{aligned} & \bigl\vert A\bigl(a^{n},b^{n} \bigr)-L_{n}^{n}(a,b) \bigr\vert \leq\frac{n(b-a)}{4}A \bigl( \vert a \vert ^{n-1}, \vert b \vert ^{n-1}\bigr). \end{aligned}$$
